# Myocardial deformation (strain) measured by DENSE reliably detects myocardial scar

**DOI:** 10.1186/1532-429X-14-S1-P243

**Published:** 2012-02-01

**Authors:** Johan Kihlberg, Henrik Haraldsson, Tino Ebbers, Jan E Engvall

**Affiliations:** 1 CMIV, Linkoping University, Linkoping, Sweden; 2 IMH / Clinical Physiology, Linkoping University, Linkoping, Sweden; 3 Radiology department, Linkoping Univeristy Hospital, Linkoping, Sweden

## Summary

The purpose of this study was to explore the differences in myocardial deformation by DENSE determined in healthy myocardium and in segments variously affected by myocardial infarction. There was a significant difference in both radial and circumferential strain between normal and infarcted myocardium.

## Background

The purpose of this study was to explore the differences in myocardial deformation by DENSE determined in healthy myocardium and in segments variously affected by myocardial infarction. There was a significant difference in both radial and circumferential strain between normal and infarcted myocardium.

## Methods

By using a 1.5T scanner have 68 patients with a high likelihood of coronary artery disease been included. The following MRI protocol was used: cine SSFP, single slice multiphase Displacement ENcoding with Stimulated Echoes (DENSE) during breath holding, perfusion during adenosine stress and at rest and late gadolinium enhancement. Ejection fraction was calculated from the short axis cine and wall motion was assessed in terms of normal, hypokinetic, akinetic and dyskinetic according to guidelines. Perfusion was assessed as normal or reduced in parts of the thickness of the wall. Late gadolinium enhancement was also graded in terms of transmurality (scar percentage of the wall thickness). All measurements were applied to segments of the AHA seventeen segment model, excluding the apical cap. DENSE post processing was performed with an in-house developed software calculating radial strain and circumferential strain. Healthy myocardium was determined if scar was absent and, wall motion and perfusion were normal (n=11). Significant myocardial infarction was defined as positive gadolinium enhancement in excess of fifty percent wall thickness (n=27). Segments were aggregated according to the coronary perfusion territories.

## Results

**Figure 1:**
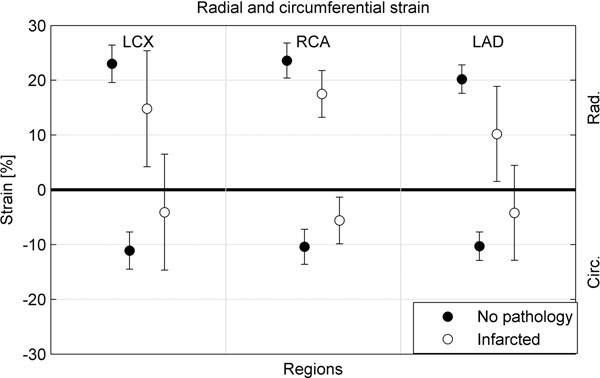


Data are shown for healthy/scared areas (p<0.0005). LCX area: radial strain 23% / 15%, circumferential strain was -11%/ -4% RCA area: radial strain 24% / 18%, circumferential strain -10% / -6%. LAD area: radial strain 20% / 10% and circumferential strain -10% / -4%. The means and the standard deviation is shown in fig.1. The standard deviation in the strain values for the infarcted vessel areas depend on the added segments and the 50% cut-off value for transmurality.

## Conclusions

Myocardial infarction more than 50% transmurality can be identified by strain measurements using DENSE.

## Funding

Grants were given from County Council of Östergötland, Heart Foundation, Swedish Heart-Lung Foundation and Swedish Research Council.

